# Hydatiform Pregnancy

**Published:** 1886-02

**Authors:** E. J. Doering

**Affiliations:** 2406 Prairie Avenue


### REPORT OF A CASE OF HYDATIFORM PREGNANCY.

*By* E. J. DOERING, M. D.

[Read before the Chicago Gynæcological Society, Jan. 15th, 1886.]

Hydatiform pregnancy, or more correctly designated cystic degeneration of the villi of the chorion, is of such rare occurrence and possesses so much clinical interest, that a report of a case of the kind which recently occurred in my practice, will, I am confident, be of interest to the Fellows of this Society.

The etiology of this affection is still unsettled. Virchow attributed it to a morbid condition of the decidua; others to a morbid maternal condition, while Hewitt believes it follows the death of the fœtus, from whatever cause, the chorion remaining attached to the decidua, and stimulated by the "energy of growth" developing into a vesicular mole. This latter view has some foundation in the fact, that as a rule no trace of the fœtus can be discovered.

"The degeneration of the villi of the chorion generally commences at an early period of pregnancy, before the placenta has commenced to form. The epithelium of the villi appears to be the part first affected, and the whole interior of the diseased villi becomes filled with cells. The connective-tissue of the villus undergoes a remarkable proliferation, and collects in masses at individual spots, the remainder of the villus being unaffected. By the growth of these elements the villus becomes distended and many of the cells liquefy, the intercellular fluid, thus produced, widely separating the connective-tissue, so as to form a network in the interior of the villus. Thus are formed the peculiar grape-like bodies which characterize the disease. When once the degeneration has commenced, the diseased tissue has a remarkable power of increase, so that it sometimes forms a mass as large as a child's head, and several pounds in weight."—(*Playfair's Midwifery.*)

The attachments of the vesicular mole to the uterus vary. Schroeder reports a case in which the vesicles penetrated throughout the uterine wall to the peritoneum, and Dr. Tyler Smith saw a similar condition, which led to a rupture of the uterus during the expulsive pains. By reason of the rapidity of the growth and the great distension of the uterus, expulsion of the mass generally occurs between the fourth and sixth months. The mass is usually expelled piece-meal, accompanied by profuse and exhausting hæmorrhages, thus exposing the patient to all the dangers of septicæmia and rendering the prognosis in any case doubtful.

The symptoms of a hydatiform pregnancy, such as rapid increase in the size of the uterus, a doughy sensation to the touch, general malaise, etc., are all uncertain and misleading, and practically it is impossible to make a correct diagnosis until portions of the vesicles are expelled, as in the following case:

Mrs. W. D. P., a cultured lady, of slender physique, 21 years of age, was attended by me in labor fifteen months ago, and delivered by instruments of a healthy boy weighing ten pounds. Her general health has been good. She has had no miscarriages either previous to or since the birth of her child. Her last period occurred during the latter part of October, 1885. During the month of November the catamenia remained absent, which she attributed to a cold, the idea of pregnancy not occurring to her, as she had none of the usual symptoms. During the month of December, and particularly during the week preceding the holidays, she was on her feet constantly, although not feeling well, having sensations of chilliness, followed by a feeling of heat and general depression. On the Sunday before Christmas a slight and painless flow of blood commenced, believed by her to be the period, now four weeks overdue. The flow continued several hours and then ceased. On Christmas day, while seated at the dinner-table, she was suddenly attacked with a profuse hæmorrhage, the blood saturating the floor, and continuing until a degree of faintness was produced, in which condition I found her on my arrival a few minutes afterwards. The hæmorrhage, which had been entirely without pain, ceased suddenly. A careful examination confirmed my suspicion of pregnancy, although I was much surprised at the size of the uterus, corresponding to a four and one-half months' pregnancy, the fundus rising nearly midway between the symphysis pubis and the umbilicus. There being no further hæmorrhage, no pain and no dilatation of the os, an expectant plan of treatment was pursued by instructing the patient to keep in bed, enjoining absolute rest, and giving her a few doses of morphia. On the following night another hæmorrhage occurred, but of not much consequence, and requiring no interference. Two days later, on the morning of the 28th of December, another hæmorrhage took place, more copious than the last one, but still unaccompanied with pain. An examination showed slight dilatation of the os, but not sufficient to permit the recognition of the contents of the uterus. As the patient was beginning to show decided symptoms of anæmia, the vagina was tamponed and ergot administered to check the hæmorrhage and favor uterine contractions.

Uterine pains soon commenced, accompanied by considerable hæmorrhage ; the os dilated fully one inch, the presenting part giving the sensation to the finger of a blood-clot. This was soon expelled in detached portions, and on removal from the vagina was readily recognized as a hydatiform mole, having all the characteristic appearances of a grape bunch, composed of a mass of translucent vesicles, about the size of currants, containing a clear limpid fluid. After inserting two fingers into the uterus and emptying it as thoroughly as possible of all the diseased tissue, the hæmorrhage promptly stopped. The entire mass removed equalled about the size of a large orange. Some febrile reaction occurred, but for several days the temperature did not exceed 100½° and the pulse 95, the treatment consisting of quinine and ergot, internally, and the use of uterine and vaginal injections of carbolized water.

On the beginning of the fourth day the patient was suddenly seized with a severe chill, followed by the usual symptoms of septic poisoning, high temperature (104½°), rapid and feeble pulse, superficial respiration, great tympanites, thirst, vomiting, and arrested lochia, with no pain or tenderness over the abdomen. The outlook was anything but promising, but the prompt administration of large doses of quinine, combined with diaphoretics, turpentine stupes, warm fomentations, and the continued use of antiseptic injections was followed by the most gratifying results, and, after four days of great anxiety, the patient had recovered sufficiently to be declared out of danger. At the present time, eighteen days since the expulsion of the mole, the patient is up and about the house, with a good appetite and making preparations to leave in a week or two on a journey to the south.

2406 Prairie Avenue.

